# Quantification of Vortex Generation Due to Non-Equilibrium Electrokinetics at the Micro/Nanochannel Interface: Particle Tracking Velocimetry

**DOI:** 10.3390/mi7070127

**Published:** 2016-07-21

**Authors:** Seung Jun Lee, Kilsung Kwon, Tae-Joon Jeon, Sun Min Kim, Daejoong Kim

**Affiliations:** 1 Department of Mechanical Engineering, Sogang University, Seoul 04107, Korea; seungjun@illinois.edu (S.J.L.); kks840214@sogang.ac.kr (K.K.); 2 Department of Biological Engineering, Inha University, Incheon 22212, Korea; tjjeon@inha.ac.kr; 3 Department of Mechanical Engineering, Inha University, Incheon 22212, Korea

**Keywords:** vortex generation, nanochannel, microparticle, particle tracking velocimetry

## Abstract

We describe a quantitative study of vortex generation due to non-equilibrium electrokinetics near a micro/nanochannel interface. The microfluidic device is comprised of a microchannel with a set of nanochannels. These perm-selective nanochannels induce flow instability and thereby produce strong vortex generation. We performed tracking visualization of fluorescent microparticles to obtain velocity fields. Particle tracking enables the calculation of an averaged velocity field and the velocity fluctuations. We characterized the effect of applied voltages and electrolyte concentrations on vortex formation. The experimental results show that an increasing voltage or decreasing concentration results in a larger vortex region and a strong velocity fluctuation. We calculate the normalized velocity fluctuation—whose meaning is comparable to turbulent intensity—and we found that it is as high as 0.12. This value is indicative of very efficient mixing, albeit with a small Reynolds number.

## 1. Introduction

Research on micro/nano fluidic systems for analyzing chemical and biological samples has dramatically increased over the past decades [1,2,3,4,5,6,7,8,9]. Various experimental studies have been performed on diverse micro/nano devices; for example, micro-total analysis systems (µTAS), sample preconcentrators, and DNA analyzers [10,11,12,13,14,15,16,17,18,19,20,21]. Rapid mixing is known to be challenging in these micro/nano fluidic systems because of the low Reynolds numbers at this small scale. Researchers have demonstrated a number of rapid mixing schemes for micro/nano fluidic systems. A promising mixing method is based on vortex formation near a micro/nano channel interface [22,23,24,25,26,27,28,29]. 

Vortex generation induced by non-equilibrium electrokinetic phenomena is related to concentration polarization (CP) and nonlinear flow across nanoporous membranes [17,30,31,32,33,34,35,36,37,38,39]. With collapsing concentration polarization phenomena, an ion concentration gradient is generated in a microchannel and subsequently generates an unstable vortex around the micro/nanochannel interface [22,23,38]. We previously reported the spectral analysis of vortex generation [39]. In this study, we calculated the temporal power spectra of vortex generation, and investigated the relationship between frequency and two major operation parameters: applied voltage and solution concentration. This work shows that a rapid vortex was generated with a higher applied voltage in a lower electrolyte concentration [39], which is in good agreement with a study showing rapid mixing with the same input parameters [23]. However, experimental studies on velocity fields near micro/nano channel interfaces and more detailed velocity analysis of microfluidic vortex generation using different approaches are needed. 

Experimental studies of vortex-generated microfluidic mixing systems have been performed. Han, et al. visualized strong vortex structures generated by non-equilibrium electrokinetic phenomena [33,34,35]. Santiago, et al. reported theoretical and experimental approaches to explain vortex generation around micro/nanochannel interfaces [36]. Kim, et al. proposed a U-shaped microfluidic device using vortices near micro/nanochannel interfaces to enable mixing [22]. Lee and Kim achieved millisecond-order rapid mixing in their microfluidic device using vortices induced by non-equilibrium electrokinetics [23].

In this study, we focused on the characterization of vortex generation. We visualized a vortex structure around micro/nano channel interfaces using a fluorescent microscopy-based particle tracking method. We report the trend of vortex generation with applied voltage and ion concentration using temporal power spectral analysis, and we approached the vortex formation characteristics in terms of particle velocity. Particle tracking velocimetry was conducted in order to quantify vortices, and we measured the particle velocity to investigate the flow instability. We show different velocity fields with varying applied voltages and concentrations of electrolytes. We also characterize the flow characteristics of non-equilibrium electrokinetics with mean velocities and instantaneous fluctuations.

## 2. Experimental

**Figure 1:**
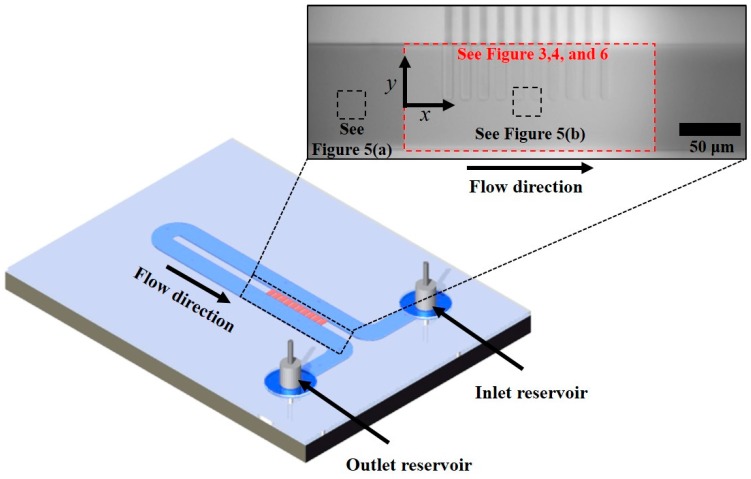
Schematic of the microfluidic device. The red rectangle indicates the region for the results in Figure 3, Figure 4 and Figure 6. The black rectangles indicate the region for the results in Figure 5.

The non-equilibrium electrokinetic microfluidic device is comprised of a U-shaped microchannel and a set of nanochannels across the two sections of this microchannel, as shown in Figure 1. The microchannel has a single stream, and there is short-circuited flow through nanochannels before and after the U-turn. The amount of fluid flow is expected to be negligible through the nanochannels because of high flow resistance. The purpose of these nanochannels is to create CP or vortex generation around the micro/nanochannel interface. The fabrication steps for this microfluidic device are described in our previous paper [39], and the configuration of the microchannel is slightly different because we applied additional pressure-driven flow in this study. 

The microchannel has a depth of 15 µm, a width of 100 µm, and an overall length of approximately 1.2 cm. The distance between the two microchannel sections—across which the set of ten nanochannels is located—is 50 µm. This distance thus coincides with the nanochannel length. All of the nanochannels are 50 nm deep and 10 µm wide. Figure 1 shows a red rectangle and two black rectangles, which indicate the regions for the results shown in Figure 3, Figure 4, Figure 5 and Figure 6. We describe the calculations for these regions below.

We used potassium chloride (KCl) solutions of 1 to 1000 mM as a working fluid. A single stream contains KCl solution with a minute amount (~0.5 µM) of fluorescent microparticles, which aids in vortex visualization. The microparticles have a nominal diameter of 2 µm. We tested smaller microparticles with diameters of 500 nm and 1 µm. These smaller microparticles emit a very low fluorescent signal below the detection limit. With our current fluorescent microscopy setup, 2 µm-sized microparticles generate a sufficient level of fluorescent signal. The surface potential of the microparticles is around 30 mV and the density of the microparticles is 1020 kg/m^3^ [40]. We maintained a low particle concentration (10 to 12 microparticles in a 100 µm × 100 µm area) for working solutions because particle tracking velocity requires sufficient spacing between particles. 

We used a power supply (IT 6834, Itech, Nanjing, China) to apply electrical power to the microfluidic device through platinum wires inserted into two reservoirs. The polarity was positive, with the same voltage at the inlet of the device, and was grounded at the outlet. We generated the pressure-driven flow using a syringe pump that was directly connected to the inlet. We performed a control experiment with a no-applied-voltage condition (only pressure-driven flow) and visualized the path lines of the microparticles. We applied voltages of 50 to 150 V while tracking the movement of microparticles. We collected images for over 100,000 microparticles in the visualization experiments, and we used MATLAB along with PC image processing tools to obtain particle tracking images.

We used an inverted epifluorescent microscope (IX-51, Olympus, New York, NY, USA) to track these particles in a dark room condition. The numerical aperture (NA) of the microscope optical lens is 0.6 and its magnification (M) is 40. The frame rate during all experimental steps was 20 frames/s. The exposure time was slightly different depending on the particle’s path line (1.25 to 1.5 s). We performed particle tracking velocimetry using the Image Pro Plus software (7.0). The particle tracking visualization gathers rather limited information compared to particle image velocimetry. Attempts at particle image velocimetry failed due to the limitations of our experimental setup. However, we successfully obtained a time-averaged velocity field and useful statistical information about the flow velocities. 

In the experiments, we completely filled the microchannels with KCl solution containing 2 µm-sized microparticles after initial flushing steps with deionized water. Then, we applied direct current (DC) electric potentials across the microfluidic device through a Pt electrode inserted in both reservoirs, and observed the micro/nanochannel interfaces using an inverted microscope. We collected images for over 100,000 microparticles passing through the micro/nanochannel interfaces and analyzed the directional velocity of each microparticle. From the directional velocity data of each particle, we plotted the velocity field on the same plane, focusing on micro/nanochannel interfacing area affected by the generated vortices. Axial movement of particles indicates the direction and magnitude of the generated vortices.

## 3. Results and Discussion 

**Figure 2:**
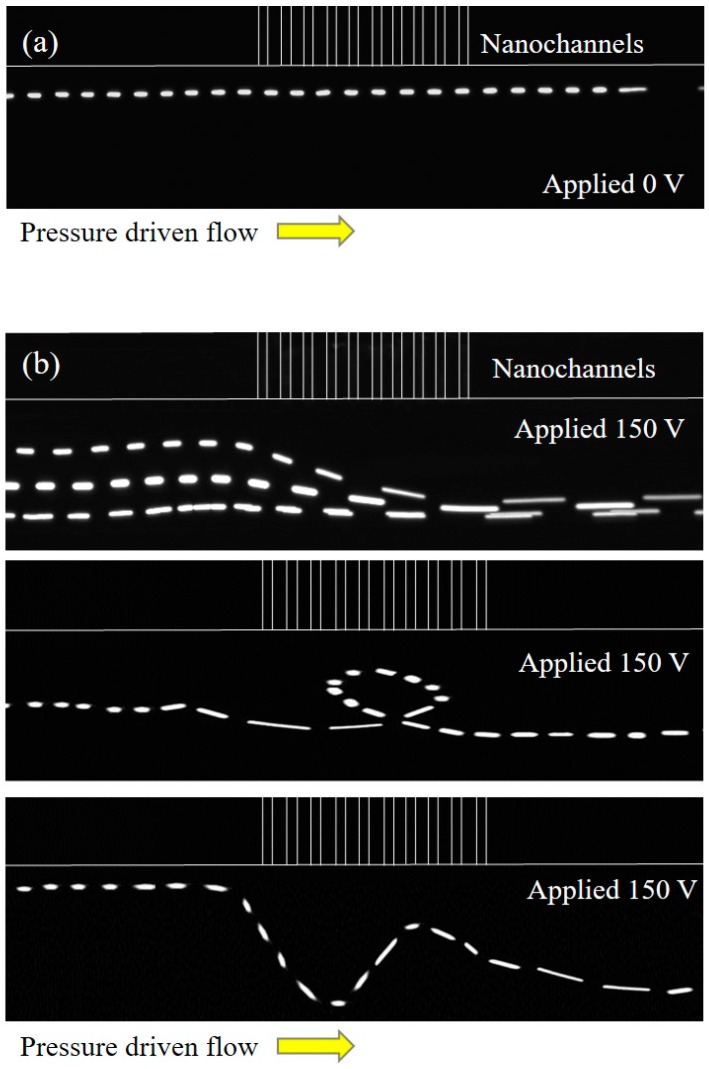
(**a**) Particle path lines with no voltage and (**b**) particle path lines with an applied voltage of 150 V.

Figure 2 shows the path lines for (a) a pure pressure-driven flow and (b) a combined electroosmotic and pressure-driven flow. For the pressure-driven flow, the flow rate is fixed at 1 µL/min. This flow rate is the same in all of the experiments performed in this study. As shown in Figure 2a, most microparticles move along the direction of the pressure drop, as expected. The particle paths are visualized as straight lines along the microchannel with nearly no distortion, even near the micro/nanochannel interface. Figure 2b shows three images of vortex generation around the nanochannel interface with an applied voltage of 150 V and a 1 mM KCl concentration. These images show different vortex structures at different instances for the same conditions. As is typically required in particle tracking velocimetry, we maintained a low concentration of microparticles in the solution to track the correct path line of the microparticles. In Figure 2b, the upper image of the vortex generation shows that particles can be depleted from the micro/nanochannel interface because of vortices. The middle image in Figure 2b shows that microparticles can be swirled toward the nanochannels. The lower image of Figure 2b shows the generation of multiple vortices near the micro/nano channel interface. These three distinct path line types indicate an almost random nature of vortex generation at the nanochannel interface. 

**Figure 3:**
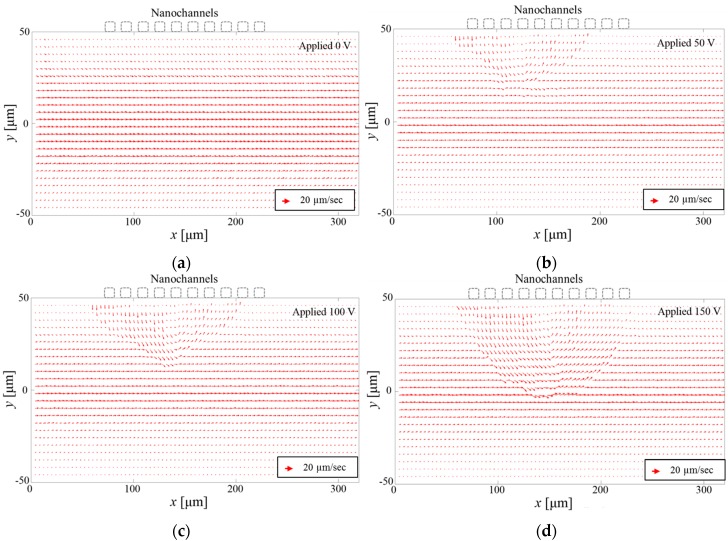
Velocity field particle tracking with (**a**) no applied voltage; (**b**) 50 V; (**c**) 100 V; and (**d**) 150 V.

We calculated the instantaneous velocities of all the microparticles from the particle traces, based on the difference in the particle displacement between the consecutive time steps. We binned them in the flow region indicated by the red rectangle in Figure 1. We then averaged the velocities of different microparticles in each bin. Figure 3 shows the time-averaged velocity field near the micro/nano channel interface for various applied voltages. The ion concentration of the solution is fixed at 10 mM. The velocity field for the pure pressure-driven flow (zero applied voltage) shows a typical Poiseuille flow profile in the microchannel. We could not find a noticeable flow distortion, even near the nanochannel interface, meaning that negligible flow occurred through the nanochannels. This is understandable from the fact that the nanochannel cross-sectional area (50 nm in depth) is very small compared to the microchannel area (15 µm in depth). On the contrary, the velocity field is distorted near the micro/nanochannel interface with an electric voltage applied. As the applied voltage is increased from 50 to 150 V, the velocity field distortion becomes more significant near the nanochannel interface, and this vortex region became larger, as shown in Figure 3. This behaviour indicates the effect of the increased applied voltage on wider vortex generation. We report this effect quantitatively, whereas previous studies considered the same physics qualitatively. We suggest the possibility of controlling the size of a mixing region in active microfluidic mixers by using a non-equilibrium electrokinetic mixing mechanism, and we believe that our quantitative velocity field measurement could be useful in future mixer development.

**Figure 4:**
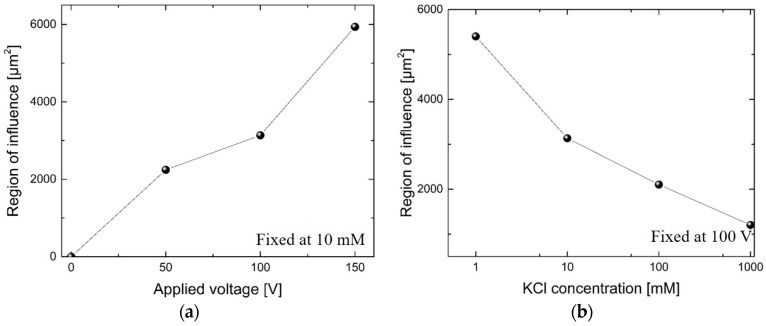
Region of influence of vortex generation versus (**a**) applied voltage and (**b**) KCl concentration.

We quantified the size of a vortex region (a region of vortex influence) as follows. (1) We first subtracted the velocity field of a pure pressure-driven flow from the velocity field of combined electroosmotic and pressure-driven flows; and (2) we summed up the area of the nonzero bins between these two fields. Figure 4 shows plots for the region of influence compared to the pure pressure-driven flow while we varied (a) applied voltages; and (b) electrolyte concentrations. The size of this region of influence shows how wide vortices are generated. It could also be indicative of the strength of electrokinetic instability, which is often a result of vortex generation [22]. These results show that a greater applied voltage generates a wider region of influence. This is consistent with the strong vortex formation results with an increased applied voltage from previous research [23]. Results with varying concentrations of electrolyte show that a larger vortex region forms with a lower concentration. Vortex generation is known to be stronger with a smaller nanochannel depth relative to the Debye length, which is inversely proportional to ion concentration [22]. In this work, the Debye length—ranging between 0.5 and 15 nm—increased with decreasing concentration, while the nanochannel depth was fixed. Vortex generation is therefore stronger with a lower concentration. This result is consistent with the results of our previous study, wherein we reported improved mixing performance with lower concentration in a microfluidic mixer [23].

**Figure 5:**
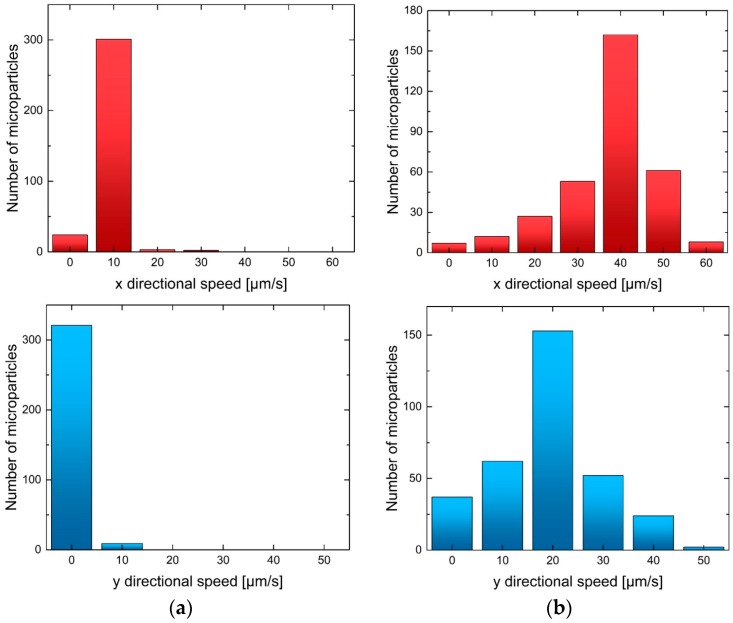
Speed distribution in (**a**) a non-vortex region and (**b**) a vortex region (see Figure 1).

From the particle tracking images, we also calculated velocity fluctuations, in addition to the averaged flow velocities reported thus far. The applied voltage and ion concentration are fixed at 100 V and 10 mM, respectively. We show two regions (the black rectangles in Figure 1) to compare the vortex generation: (a) shows the region where vortices are almost absent; and (b) shows the region where vortices are strong. Figure 5 shows the distribution of the instantaneous speed (the absolute value of the velocity) of microparticles in two regions. The *x*- and *y*-directional speed distributions in Figure 1a show small speed fluctuations, confirming the absence of vortex generation. Especially, the *y*-directional speed is almost zero, indicating that there is no lateral motion of microparticles. On the contrary, the speed distribution in Figure 1b shows the scattering of the data in both the directions, thereby confirming the existence of vortices. Both distribution profiles show a unimodal distribution with maximum values of 40 m/s and 20 µm/s for the *x*- and *y*-directions, respectively. The *y*-directional speed is nonzero near the micro/nano channel interface, which explains the diverse particle traces shown in Figure 2.

To see the effect of the operating conditions on this velocity fluctuation, we calculated the normalized velocity fluctuations averaged over the region indicated by the red rectangle shown in Figure 1. We varied the applied voltages and KCl concentrations in our experiments. The calculated normalized velocity fluctuation is comparable to turbulent intensity, which is a useful indicator of flow instability. The turbulent intensity (which is often referred to as the turbulence level) is defined as $I=u^{\prime }/U$, where $u^{\prime }$ is the root-mean-square (RMS) of the turbulent velocity fluctuations and *U* is the mean velocity [41]. Our normalized velocity fluctuation is clearly different from this turbulent intensity, because the RMS of the velocity fluctuations are calculated with respect to the *x*- and *y*-directions, not all three directions. In our experiments, we were not able to obtain an out-of-plane velocity component without the use of confocal microscopy. 

**Figure 6:**
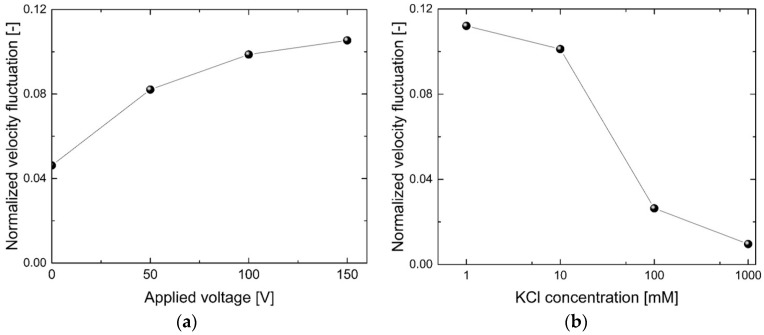
Normalized velocity fluctuation versus (**a**) applied voltage and (**b**) KCl concentration.

Figure 6 shows the normalized velocity fluctuations with varying voltages and concentrations. We obtained the normalized velocity fluctuation up to 0.12 (its value is over 0.05 for most data points). The Reynolds number is estimated to be 0.056, based on the hydraulic diameter and the electroosmotic velocity. This low Reynolds number should mean that laminar flow occurred in the microchannel. However, our calculated velocity fluctuation shows a significant level of fluctuation, indicating that our device can be useful for efficient microfluidic mixing.

Figure 6 also shows that a higher applied voltage or a lower electrolyte concentration generates stronger velocity fluctuations. This result is consistent with the size of the vortex region shown in Figure 4. The conditions for vortex generation are that the nanochannel depth should be of a similar order of magnitude to the Debye length (varying with different KCl concentrations), and that the applied voltage should be high enough to be in the over-limiting current regime [34,38]. Vortex generation phenomena are based on the CP effect, and vortex generation occurs when the concentration in the depletion region is very low (i.e., a relatively high electric field exists in this region). This condition in the depletion region causes all of the electrolyte to have a very high electrophoretic velocity. The condition can be amplified at the micro/nanochannel interface area, and it results in large concentration gradients in the background electrolyte. The steep change in concentration generates high electric field gradients. These electric field gradients create vortices near the micro/nanochannel interface, especially on the depletion side. The velocity of the electrolytes finally results in flow instability, which we observed in our experiments. 

Regarding Joule heating, we measured the temperature of the reservoirs with the highest applied voltage and the highest concentration. The increase in temperature is insignificant, and we think that the short length of the microchannel and the large size of the reservoirs mitigate the effect of Joule heating. 

## 4. Conclusions

We investigated non-equilibrium electrokinetic vortex generation near a micro/nanochannel interface using the microparticle tracking method. Particle tracking images were useful in obtaining average velocity fields and velocity fluctuations. We observed the effect of the applied voltage and the electrolyte concentration on vortex generation. The average velocity fields confirm that vortex generation is strong near the micro/nanochannel interface. We found that the region of strong vortices becomes larger with increasing voltage or decreasing concentration. The observed normalized velocity fluctuations are consistent with the result that the increasing trend of velocity fluctuations is related to the size of the vortex region. We note that the value of the normalized velocity fluctuation—which is comparable to turbulence intensity—is up to 0.12. This indicates strong vortices and thus efficient mixing in a microfluidic platform.
